# Reconceptualizing negative endoscopy as a positive contextual indicator for disorders of gut-brain interaction

**DOI:** 10.3389/fphys.2026.1795494

**Published:** 2026-03-12

**Authors:** Wumiao Zhang, He Zeng, Qian-Nan Ruan, Dakai Zeng

**Affiliations:** 1 The First Affiliated Hospital of Gannan Medical University, Ganzhou, Jiangxi, China; 2 Third Affiliated Hospital of Wenzhou Medical University, Wenzhou, Zhejiang, China; 3 Wenzhou Seventh People's Hospital, Wenzhou Medical University, Wenzhou, Zhejiang, China

**Keywords:** brain-gut axis, disorders of gut-brain interaction (DGBI), GI endoscopy, physician-patient communication, visceral hypersensitivity

## Abstract

In the contemporary landscape of gastroenterology, the “negative” endoscopy—an examination revealing no gross structural, mucosal, or biochemical abnormalities—is frequently perceived by both clinicians and patients as a diagnostic non-event, a failure to identify a cause, or an ambiguous dead-end. This perspective paper argues for a fundamental epistemological and clinical paradigm shift: a negative endoscopic result, when combined with appropriate biopsy protocols and clinical history, should not be classified merely as the absence of organic disease but as positive, corroborative evidence acting as a contextual indicator supporting a diagnosis of a Disorder of Gut-Brain Interaction (DGBI). By integrating the verified absence of macroscopic “hardware” damage (structural pathology) with the presence of characteristic symptom patterns, clinicians can support the diagnosis of a “software” malfunction (visceral hypersensitivity and altered central processing). This report provides a detailed analysis of the physiological mechanisms of the brain-gut axis that underpin this argument, dissects the psychological impact of diagnostic labeling versus invalidation, and proposes a comprehensive novel clinical workflow—the “Affirm-Explain-Transform” (AET) model—to convert the negative endoscopy from a source of clinical ambiguity into a potent therapeutic tool.

## 1 Introduction

Gastrointestinal (GI) endoscopy has evolved into the cornerstone of modern gastroenterological practice. However, its utility in evaluating common symptoms like dyspepsia and abdominal pain faces increasing scrutiny (Moayyedi et al., 2017). Regarding “uninvestigated dyspepsia”—a condition with a global prevalence estimated at approximately 21%—data reveals a stark discrepancy between the volume of testing and the yield of organic pathology (Ford et al., 2015). Extensive epidemiological studies and clinical guidelines demonstrate that in patients undergoing EGD for dyspepsia, up to 70%–80% will have no structural abnormalities detected (Ford et al., 2010). Similarly, colonoscopies performed for non-alarm symptoms frequently return “normal” results, ruling out inflammatory bowel disease or neoplasia but leaving the patient’s debilitating symptoms unexplained (Asghar et al., 2022).

This creates a pervasive clinical paradox. While the procedure is a technical success that rules out life-threatening conditions, the result “Everything is normal” stands in jarring contradiction to the patient’s lived experience of pain. This discordance often generates a crisis of validation, implying to the patient that their symptoms are “not real” or “all in the head”. Historically, Functional Gastrointestinal Disorders (FGIDs) were conceptualized as “diagnoses of exclusion,” labels applied only after a battery of tests returned negative. This framework drives inefficient healthcare utilization and massive economic burden; recent updates indicate that the economic burden of gastrointestinal diseases in the US remains immense, with functional disorders contributing significantly to these expenditures (Peery et al., 2022). Consequently, this exclusion model leaves patients in a state of “illness uncertainty,” which is highly correlated with anxiety and poor quality of life.

The introduction of the Rome IV criteria marked a pivotal turning point, recharacterizing these conditions as Disorders of Gut-Brain Interaction (DGBI) (Drossman, 2016). These are defined by positive pathophysiological mechanisms, including motility disturbance, visceral hypersensitivity, and altered central nervous system processing (Schmulson and Drossman, 2017). In this era, the role of endoscopy is recontextualized; a negative endoscopy definitively helps rule out macroscopic luminal mucosal pathology (“hardware”). Therefore, provided extra-luminal causes are unlikely, a pristine mucosa serves as specific, corroborative evidence that the organ structure is intact, thereby localizing the pathology to the “software”—the neuro-regulatory and sensory processing systems (Drossman, 2016). This perspective posits that a negative endoscopic result functions as a clinical indicator for DGBI, allowing clinicians to pivot from diagnostic testing to targeted therapy addressing the gut-brain axis (Black et al., 2022).

### 1.1 Diagnostic boundary conditions

It is essential to state explicitly that the relationship between a negative endoscopy and a DGBI diagnosis is corroborative, not purely deterministic. A negative esophagogastroduodenoscopy (EGD) or colonoscopy serves as supportive evidence only within an appropriate clinical context and after the reasonable exclusion of alternative etiologies. Standard endoscopies primarily evaluate the proximal gastrointestinal tract and the colon, leaving the majority of the small bowel unevaluated. Therefore, small bowel pathologies beyond the reach of standard endoscopes (e.g., obscure Crohn’s disease, small intestinal bacterial overgrowth) must remain in the differential diagnosis. Furthermore, a pristine mucosa does not exclude metabolic or endocrine dysfunctions (e.g., diabetes mellitus, thyroid disorders), pancreaticobiliary diseases, or medication-induced gastrointestinal symptoms. Thus, reframing a negative endoscopy as a positive contextual indicator is valid only when extra-luminal, systemic, and pharmacological causes have been adequately assessed and excluded through a comprehensive clinical history and fundamental evaluations.

## 2 Physiological mechanisms of the brain-gut axis: an integrative review

To validate negative endoscopy as a positive contextual indicator, the biological basis of symptoms in Disorders of Gut-Brain Interaction (DGBI) must be established. Historically, the biomedical model equated “disease” with visible structural pathology; however, modern neurogastroenterology has delineated the Brain-Gut Axis (BGA), demonstrating that DGBIs involve physiological dysregulation operating at a neuro-molecular level not visible via standard white-light endoscopy (Mayer, 2011).

### 2.1 The brain-gut axis

The Brain-Gut Axis constitutes a complex, bidirectional network linking the central nervous system (CNS) and the enteric nervous system (ENS) via neural, hormonal, and immunological pathways. The ENS is frequently characterized as the “second brain” due to its autonomy and complexity, comprising approximately 500 million neurons, a quantity comparable to the spinal cord (Furness, 2012). In homeostasis, this axis operates largely below the threshold of conscious perception, transmitting sensory data regarding mechanical distension and chemical environment while regulating motility and secretion. In DGBIs, this communication is maladaptive, with pathology located primarily in signal processing rather than the signal source. This dysregulation is driven by specific mechanisms, most notably visceral hypersensitivity and central sensitization. Visceral hypersensitivity, a hallmark of functional dyspepsia and IBS, is characterized by hyperalgesia (lowered pain threshold) or allodynia (pain from non-painful stimuli); for instance, physiological gastric distension inducing satiety in healthy individuals may be perceived as pain or unbearable fullness in patients with DGBIs (Enc et al., 2017). Concurrently, central sensitization occurs when nociceptive pathways in the spinal cord and brain become hyperexcitable. Functional MRI (fMRI) studies indicate that patients with DGBIs demonstrate distinct activation patterns in pain-processing regions, including the anterior cingulate cortex, insula, and thalamus, even in the absence of peripheral inflammation (Mayer et al., 2022). Furthermore, cognitive and emotional factors modulate visceral signaling, as stress and anxiety amplify signal intensity through the dysregulation of the HPA axis and autonomic nervous system (Mayer et al., 2015).

By integrating these well-established mechanisms, we can construct an explicit physiological model for interpreting diagnostic testing: the physiological utility of a negative endoscopy lies in its capacity to actively localize the pathological defect. When macroscopic structural damage and gross luminal inflammation are definitively excluded, the negative finding serves as a physiological filter. It shifts the anatomical focus of symptom generation away from peripheral tissue destruction and functionally localizes the pathology to the neuro-regulatory network—specifically, altered mechanosensory thresholds in the ENS and descending modulatory failures in the CNS. Thus, a macroscopically normal mucosa acts not as a void of information, but as specific corroborative evidence isolating the functional defect to brain-gut signaling. The key differences between the traditional ‘diagnosis of exclusion’ approach and our proposed ‘positive contextual indicator’ framework are summarized in Table 1.

**TABLE 1:** Comparison of the traditional vs. proposed diagnostic model.

Feature	Traditional “exclusion” model	Proposed “positive contextual indicator” model
View of negative scope	“Nothing is wrong”; diagnostic failure	“Luminal hardware is intact”; corroborates DGBI diagnosis
Primary goal	Rule out cancer/organic disease	Exclude macroscopic structural damage to corroborate neuro-functional dysfunction
Patient communication	“Good news, it’s normal.” (implies dismissal)	“The tissue is healthy, which directs us to treat the nerve sensitivity (software)”
Next steps	Discharge or repeat testing for other causes	Review biopsy; if negative, initiate DGBI management immediately
Patient outcome	Uncertainty, anxiety, doctor shopping	Validation, acceptance, adherence to therapy

### 2.2 The “hardware vs. software” analogy

To explain why endoscopy is negative in these conditions despite genuine pathology, the “Hardware vs. Software” metaphor is clinically invaluable and scientifically grounded (Drossman, 2016). The “Hardware” (Structure) refers to the physical integrity of the GI tract—the mucosa, muscle layers, and gross anatomy. Diseases like peptic ulcers, tumors, diverticulitis, or inflammatory bowel disease represent “hardware damage” where the tissue is physically broken or inflamed. Endoscopy is a visual inspection tool designed specifically to audit this hardware. Conversely, the “Software” (Function/Processing) represents the electrical signaling, chemical messengers, and data processing that occur between the ENS and CNS. DGBIs can be conceptualized as “software glitches” where the physical nerves and muscles are intact, but the signals they send are scrambled, amplified, or misinterpreted (Drossman and Ruddy, 2020). For example, a “software” error might cause the stomach to signal “fullness” after only a few bites or the colon to signal “urgent evacuation” upon mild distension. In this framework, when an endoscopist confirms the physical machinery is intact, the negative result serves as corroborative clinical data supporting the “software” diagnosis. It helps rule out the competing hypothesis of macroscopic hardware damage, making the DGBI diagnosis an appropriate physiological inference rather than a mere diagnosis of exclusion (Drossman, 2016).

### 2.3 Micro-inflammation and the neuro-immune interface zone

It is critical to clarify that the “hardware vs. software” dichotomy is a clinical heuristic framework intended for patient-physician communication, not a strict ontological separation. This boundary is notably blurred at the neuro-immune interface by findings such as low-grade duodenal inflammation in functional dyspepsia (Talley and Ford, 2015) or microscopic colitis, which present with macroscopically normal mucosa but distinct histological pathology. Therefore, the definition of a “negative endoscopy” in the context of DGBI diagnosis implies both macroscopic and microscopic clearance. While standard endoscopy may label the mucosa “normal,” subtle immune activation—such as increased mucosal permeability or low-grade mast cell infiltration—represents microscopic structural pathology. However, rather than invalidating the functional model, these findings highlight a biological bridge: mediators released in this neuro-immune interface zone directly interact with enteric nerves to lower the pain threshold (Barbara et al., 2004; Huyghe et al., 2025). Thus, micro-structural “hardware” alterations generate “software” signaling errors. Consequently, a “negative” macro-endoscopy remains the crucial filter, ensuring that the treatment pathway focuses on neuromodulation and microbiome management therapies targeting the micro-environment and signaling, rather than surgical interventions required for gross structural disease 8.

## 3 The psychology of diagnosis—communication and the placebo/nocebo effect

The second pillar of the argument for considering negative endoscopy as corroborative evidence lies in the domain of communication and psychology. How a test result is delivered is as clinically potent as the medication prescribed. In the context of DGBI, the handling of negative results has historically been iatrogenic—causing harm through invalidation.

### 3.1 The “all clear” paradox and the nocebo effect

When a patient wakes up from sedation, the common script “Good news, everything is normal” can be devastating for a symptomatic patient. This message creates cognitive dissonance (“If everything is normal, why am I in pain?”), contributing to a specific form of the nocebo effect where the “harm” is the invalidation of the patient’s suffering (Colloca and Miller, 2011). The implication that “nothing is wrong” leads the patient to internalize the problem as a personal failing or psychiatric defect. Research shows that patients with “medically unexplained symptoms” experience higher levels of anxiety and lower quality of life compared to those with clear organic diagnoses, even if the organic diagnosis is chronic (Henningsen et al., 2003). The uncertainty of not knowing the cause is a potent psychosocial stressor that fuels the sympathetic nervous system and activates the hypothalamic-pituitary-adrenal (HPA) axis; since this sympathetic hyperarousal inhibits physiological digestion and lowers visceral pain thresholds, the anxiety caused by a lack of diagnosis biologically worsens the DGBI symptoms (Mayer, 2011).

### 3.2 Anxiety, intolerance of uncertainty, and the failure of reassurance

The assumption that a negative endoscopy provides lasting reassurance is challenged by data. While there is a transient reduction in anxiety immediately post-procedure, this effect often dissipates within weeks if symptoms persist without explanation (Van et al., 2016). Individuals with high Intolerance of Uncertainty (IU) are particularly vulnerable; for these patients, a negative result that does not provide a positive explanation leaves the uncertainty loop open, leading to symptom hypervigilance which further lowers the visceral pain threshold via central sensitization (Keefer et al., 2005). Additionally, “doctor shopping” emerges as a direct behavioral consequence of this failure. Patients who feel dismissed will seek another endoscopist, inflating healthcare costs and exposing themselves to procedural risks. The economic burden of negative endoscopies is driven largely by this cycle of repeat investigation due to inadequate initial diagnostic framing (Peery et al.).

### 3.3 The therapeutic value of a positive label

Conversely, providing a positive diagnosis (“Labeling”) has intrinsic therapeutic value. Validating the symptoms as a recognized condition (DGBI) facilitates Cognitive Restructuring, allowing the patient to shift from a “mystery” mindset (catastrophizing about missed cancer) to a “management” mindset (Kaptchuk et al., 2008). Furthermore, a confident diagnosis enhances the physiological placebo component of subsequent therapies. When a clinician prescribes treatment for a specifically named condition, validation and cognitive restructuring engage the brain’s descending pain inhibitory pathways (e.g., involving the periaqueductal gray), stimulating endogenous mechanisms to physically dampen ascending nociceptive signals. Therefore, the negative endoscopy should trigger immediate “positive labeling,” reframing the negative test as corroborative clinical data needed to support the DGBI diagnosis (Drossman and Ruddy, 2020).

## 4 The “affirm-explain-transform” (AET) diagnostic workflow

To operationalize this perspective, we propose a restructured clinical workflow. This model integrates the physiological localization of “software” issues with the psychological necessity of validation. It transforms the negative endoscopy from a discharge event into an admission event for DGBI management.

### 4.1 Phase 1: pre-endoscopy framing

Clinical intervention begins well before the endoscopic procedure; the pre-procedure consultation serves as a pivotal moment for setting expectations and framing potential outcomes (Van et al., 2016). A significant challenge in current clinical practice is that informed consent discussions tend to focus disproportionately on procedural risks (e.g., perforation, bleeding) and the objective of identifying pathological changes (e.g., ulcers, cancer); consequently, patients often experience disappointment if no such lesions are detected (Moayyedi et al., 2017). To address this issue, clinicians must explicitly introduce Disorders of Gut-Brain Interaction (DGBI) as a differential diagnosis on equal footing with organic disease prior to the procedure (Drossman, 2016). Clinicians may employ a narrative such as: “We are performing this endoscopy to evaluate the ‘hardware’ of your stomach. There are two primary possibilities: first, we identify a structural issue, such as an ulcer, which we will treat; second, we find that the gastric mucosa is entirely healthy. If the mucosa is healthy, this supports the premise that your pain originates from sensitive nerve endings—a ‘software’ issue known as a Disorder of Gut-Brain Interaction. Both scenarios represent real medical conditions and are treatable. Therefore, a ‘normal’ test result actually serves as a positive contextual indicator of sensitive nerve-based pain” (Drossman, 2016). This form of priming ensures that in the event of negative findings, the patient is prepared to interpret a DGBI diagnosis as a corroboration rather than a dismissal, thereby preventing feelings of invalidation 6. This redefines the concept of “success,” shifting it from the detection of a tumor to the discovery of an answer (Moayyedi et al., 2017).

### 4.2 Phase 2: the new endoscopy report

The endoscopy report serves as both a medical-legal document and a critical communication tool (Cohen and Pike, 2015). Currently, standard reporting language often undermines the diagnosis of Disorders of Gut-Brain Interaction (DGBI) by relying on terms like “Negative,” “Normal,” or “Unremarkable” (Cohen and Pike, 2015). In contrast, a DGBI-oriented report frames the findings as supportive clinical data rather than a diagnosis of exclusion (Drossman, 2016). Such a report might state: “Macroscopically normal mucosa. Biopsies taken to exclude microscopic pathology. In the absence of histological or extra-luminal findings, the macroscopic normality of the ‘hardware’ is consistent with a functional etiology for the patient’s symptoms (e.g., DGBI)” (Drossman and Ruddy, 2020). The clinical significance of this shift is threefold. For the Referring Physician, it provides a clear direction to initiate management for FD/IBS rather than pursuing further unnecessary investigations (Drossman and Ruddy, 2020). For the Patient, documenting the diagnosis in “black and white” provides psychological validation, confirming that the procedure yielded a meaningful answer rather than a mere lack of findings (Drossman and Ruddy, 2020). For Insurers and Systems, this approach justifies the procedure as a supportive diagnostic step, framing the encounter as clinically productive (Drossman, 2016).

### 4.3 Phase 3: the management bridge

The endoscopic confirmation of macroscopic mucosal integrity necessitates the immediate activation of a DGBI-specific management protocol, superseding the traditional discharge model reliant on “as needed” prescriptions. Clinical education functions as the primary intervention, employing the structural-functional dichotomy to visualize neuro-regulatory mechanisms and facilitate patient understanding. Regarding pharmacological and dietary interventions, while acid suppression addresses potential reflux overlap, phenotype-specific dietary modulation—such as the Low FODMAP regimen for patients with overlapping IBS—targets luminal precipitating factors (Black et al., 2022; Serra et al., 2025).

Central to this therapeutic architecture is the introduction of neuromodulators, specifically low-dose tricyclic antidepressants. Clinicians must recontextualize these agents, dissociating them from psychiatric classifications to frame them as “visceral analgesics” or “gut-brain modulators” designed to downregulate sensory thresholds and enhance descending pain inhibition, thereby reinforcing the neural hypersensitivity hypothesis immediately following negative endoscopic findings (Drossman et al., 2018). For cases exhibiting high symptom severity or elevated anxiety, the integration of psychogastroenterology directly addresses central processing dysregulation; physiologically, this constitutes targeted behavioral training that actively rewires maladaptive neural circuits and restores autonomic balance, distinct from general psychiatric care (Keefer et al., 2022).

## 5 Future directions

The definition of Disorders of Gut-Brain Interaction (DGBI) must continue to evolve from a diagnosis of exclusion to one characterized by distinct physiological mechanisms, including visceral hypersensitivity, central sensitization, and motility disturbance. In this updated paradigm, a negative endoscopy serves not as a diagnostic endpoint or a failure to identify disease, but as a pivotal clinical milestone that effectively helps rule out macroscopic structural “hardware” pathology while corroborating the diagnosis of “software” neuro-regulatory dysfunction. To operationalize this shift, professional societies such as the ASGE and ESGE should prioritize the development of standardized reporting templates that incorporate constructive diagnostic language for symptomatic patients with normal findings. Concurrently, gastroenterology fellowship curricula require the integration of psychogastroenterology and communication strategies to effectively convey the “hardware/software” analogy, while public awareness initiatives are necessary to reframe “normal” results as a validation of a treatable sensitivity disorder rather than a dismissal of symptoms. Future technological advancements, such as mucosal impedance testing and “smart scopes,” promise to bridge the gap between structure and function by objectively quantifying physiological dysfunction during standard examinations (Vaezi and Choksi, 2017). Ultimately, clinical practice must move beyond treating the mucosa to treating the patient, interpreting the absence of structural lesions as a highly consistent indicator of brain-gut axis dysregulation and a mandate for targeted therapeutic intervention.

## Data Availability

The original contributions presented in the study are included in the article/supplementary material, further inquiries can be directed to the corresponding author.
